# Mediating effect of moral sensitivity in the relationship between mental workload and patient privacy protection among operating room nurses in China: a cross-sectional study

**DOI:** 10.3389/fmed.2025.1710268

**Published:** 2025-12-16

**Authors:** Ruyi Tan, Chang Yang, Qin Zeng, Xin Han, Yunjuan Hu, Jing Yang, Hongli Ma

**Affiliations:** Department of Anesthesiology, Chongqing University Cancer Hospital, Chongqing, China

**Keywords:** patient privacy protection, mental workload, moral sensitivity, mediation effect, operating room nurses, China

## Abstract

**Background:**

Patient privacy protection is a fundamental ethical obligation in healthcare, particularly in operating rooms where anesthesia and complex surgical procedures increase patients’ vulnerability. Mental workload and moral sensitivity have been identified as key factors influencing ethical behavior, yet their interrelationships in the context of privacy protection among operating room nurses remain unclear.

**Objectives:**

To investigate the associations between mental workload, moral sensitivity, and patient privacy protection among operating room nurses in China, and to examine the mediating role of moral sensitivity in this relationship.

**Method:**

A cross-sectional study was conducted from May to July 2025 in tertiary hospitals across Chongqing, Beijing, Harbin, and Sichuan Province. Using convenience sampling, a total of 573 valid responses from operating room nurses were analyzed using the Patient Privacy Scale, the Moral Sensitivity Questionnaire-Revised Chinese Version, and the NASA Task Load Index. Pearson correlation and mediation analyses were performed using SPSS 26.0 with PROCESS macro (Model 4), applying bootstrapping with 5,000 resamples to estimate indirect effects.

**Results:**

The mean scores for patient privacy protection, moral sensitivity, and mental workload were 82.384 ± 5.169, 45.775 ± 6.053, and 45.450 ± 14.826, respectively. Mental workload was negatively correlated with both patient privacy protection (*r* = −0.655, *p* < 0.001) and moral sensitivity (*r* = −0.575, *p* < 0.001), while moral sensitivity was positively correlated with patient privacy protection (*r* = 0.506, *p* < 0.001). Mediation analysis indicated that moral sensitivity partially mediated the association between mental workload and privacy protection, accounting for 14.98% of the total effect.

**Conclusion:**

Greater mental workload is associated with lower moral sensitivity and lower patient privacy protection among operating room nurses. Moral sensitivity partially mediates this relationship, underscoring the need for interventions aimed at alleviating workload and strengthening moral sensitivity to enhance privacy protection practices.

## 1 Introduction

The operating room (OR) represents a unique and high-pressure environment where anesthetized patients are particularly vulnerable and unable to protect themselves, placing high ethical demands on OR nurses to safeguard patient privacy and dignity (1, 2). In this context, OR nurses face specific professional duties and operational pressures, such as patient positioning, complex surgical setup, and high-stakes team communication, which contribute to mental workload and ethical dilemmas regarding privacy exposure (1, 3). Specifically, the professional duties inherent to the OR nurse’s role, such as patient positioning for surgical access, managing complex equipment and sterile fields, and communicating within a high-stakes, fast-paced multidisciplinary team, significantly contribute to their mental workload. Concurrently, these very tasks often involve exposure of the patient’s body and handling of private information, thereby creating unique ethical dilemmas pertaining to privacy protection. Protection of patient privacy constitutes a fundamental principle of medical ethics and serves as a critical foundation for maintaining trust in the physician-patient relationship. With the continuous popularization of laws and the increasing awareness of personal rights, patients’ demand for privacy protection has also gradually increased (4). However, the insufficient protection of patient privacy may lead to ethical conflicts and even medical disputes (5, 6).

Moral sensitivity is recognized as an ability that enables nurses to identify ethical issues, recognize the impact of ethical issues, and sense the consequences of decisions in patient care (7), a process which itself requires cognitive engagement (8). Studies reported that moral sensitivity was positively associated with patient privacy protection, and stronger moral sensitivity enabled nurses to make better ethical decision-making (3, 7). Theoretically, an individual’s cognitive resources are finite (9). High mental workload consumes significant amounts of these resources, which may lead to a reduction in the cognitive capacity available for identifying ethical dilemmas, thereby negatively influencing moral sensitivity (8, 10). The interference of cognitive load on decision-making has been observed in high-stakes clinical environments (11). We therefore hypothesize that sustained high workload may impair the cognitive capacity available for ethical discernment, thereby exerting a negative influence on moral sensitivity. Nursing workload refers to the cumulative amount of nursing activities performed within a defined work period, including physical workload and mental workload (12, 13). High mental workload not only negatively affected nurses and their patients, but was also associated with missed nursing care, contributed to an increased incidence of nursing errors and patient safety incidents (14, 15). However, there is no study to date has specifically indicated the relationship among mental workload, moral sensitivity, and the protection of patient privacy.

Given the current gaps in the literature, we propose the hypothesis that mental workload may negatively associated with patient privacy protection and moral sensitivity, with moral sensitivity partially mediating the relationship between mental workload and privacy protection among operating room nurses in China. This study was conducted to examine the current status of patient privacy protection, moral sensitivity, and mental workload among operating room nurses, and explore the interconnections among these three factors and test the proposed hypotheses. The findings are intended to serve as a foundation for the development of targeted interventions designed to enhance patient privacy protection behaviors among operating room nurses and ultimately improve the quality of nursing care.

## 2 Methods

### 2.1 Study design

This study used a cross-sectional design to investigate the mediating effect of moral sensitivity on the relationship between mental workload and patient privacy protection.

### 2.2 Study setting and timeframe

This study was conducted from May to July, 2025 in tertiary hospitals across Chongqing, Beijing, Harbin, and Sichuan Province, China.

### 2.3 Participants and sampling procedure

OR nurses were recruited using a convenience sampling method. The inclusion criteria for OR nurses were as follows: (1) registered nurse; (2) aged 18 years or above; (3) over 1 year of clinical experience in operating room settings. Nurses on sick leave or vacation during the survey period were excluded. All participants were informed of the purpose of this study and agreed to participate prior to completing the e-questionnaire.

### 2.4 Sample size calculation

G*power 3.1 software (Heinreich-Heine-Universität, Düsseldorf, Germany) was used to calculate the sample size in our study (16). Based on a statistical power of 0.95, a significance level (*α*) of 0.05, an effect size (f^2^) of 0.025, and with the number of predictors of 12, a sample size of at least 522 participants were determined (17). Considering a 15% dropout rate of the online survey, we recruited a total of 620 participants in our study.

### 2.5 Data collection

Data were collected via the “Questionnaire Star” platform, an online crowdsourcing platform in China. The questionnaire link was distributed to all potential participants via WeChat, the most widely used social media application in China (18, 19). All operating room nurses used WeChat and could access the e-questionnaire. Prior to proceeding with the survey, all participants were presented with an informed consent form within the e-questionnaire; only those who provided electronic consent could access the survey questions. To ensure confidentiality and anonymity, no personally identifiable information was collected in the questionnaire, and the data were stored on password-protected servers of the platform. Invalid questionnaires with answers to all questions being identical and completed within 5 min were excluded to ensure response quality. Ultimately, 573 valid questionnaires were included for the analysis in this study.

### 2.6 Measures

#### 2.6.1 Socio-demographic characteristics

The self-designed general information questionnaire was used to collect socio-demographic variables and patient privacy protection information, including sex, age, ethnicity, education level, professional title, administrative position, duration of working, awareness of privacy regulations, and participation in patient privacy protection training.

#### 2.6.2 The patient privacy scale (PPS, operating room nurses edition)

Patient privacy protection behaviors were assessed using the Chinese version of the Patient Privacy Scale (PPS), originally developed by Ozturk et al. (20) and cross-culturally adapted by Wang et al. (21) for operating room nurses in China. This scale consists of 19 items across three dimensions: surrounding environment privacy (4 items), physical privacy (7 items), and information privacy (8 items). The items are rated on a 5-point Likert scale (1 = totally disagree to 5 = totally agree), with total scores ranging from 19 to 95; higher scores indicate better privacy protection performance. The Chinese version has demonstrated strong psychometric properties in Chinese OR nurses, with a content validity index of 0.87 and a Cronbach’s *α* coefficient of 0.982 (21).

#### 2.6.3 The moral sensitivity questionnaire-revised Chinese version (MSQ-R-CV)

Moral sensitivity was measured using the Moral Sensitivity Questionnaire-Revised Chinese Version (MSQ-R-CV), which was originally developed by Lützén et al. (22), subsequently revised (23), and then adapted into Chinese by Huang et al. (24). This 9-item instrument comprises two factors: moral responsibility and strength (5 items) and sense of moral burden (4 items). All items are scored on a 6-point Likert scale (1 = completely disagree to 6 = completely agree). Total scores range from 9 to 54, with higher scores reflecting greater moral sensitivity. The Cronbach’s *α* for the total scale in its Chinese version was 0.82 (24).

#### 2.6.4 The national aeronautics and space administration task load index (NASA-TLX)

Mental workload was assessed using the Chinese version of the NASA Task Load Index (NASA-TLX), developed by Hart et al. (25), was performed in nursing profession field by Tubbs-Cooley et al. (26), and translated into Chinese through a cross-cultural adaptation process by Liang et al. (27). This scale includes six subscales: mental demand, physical demand, temporal demand, performance, effort, and frustration. Each subscale is scored along a 100-point continuum divided into 20 equal intervals. For this study, the overall workload score was calculated as the unweighted arithmetic mean of the six subscale ratings, ranging from 0 to 100; a higher score indicates a higher level of mental workload. The Chinese version has reported a Cronbach’s *α* of 0.707 among nurses (27).

### 2.7 Data analysis

Statistical analyses were performed using SPSS Statistics 26.0 with the PROCESS macro (Version 4.3). Descriptive statistics included means and standard deviations for continuous variables and frequencies with percentages for categorical demographic variables. Pearson correlation analysis assessed bivariate relationships among patient privacy protection, mental workload, and moral sensitivity. To evaluate moral sensitivity as a mediator of the relationship between patient privacy protection and mental workload, Model 4 within the PROCESS macro was employed. To assess the robustness of this mediation model, we further included all collected demographic and organizational variables as covariates to control for their potential confounding effects. This model utilized bootstrapping with 5,000 resamples to generate 95% confidence intervals (CIs) for the indirect effect. In accordance with established mediation analysis guidelines, mediation was deemed statistically significant if the 95% CI for the indirect effect excluded zero (16). A significance level of *p* < 0.05 was applied for all statistical tests. Furthermore, we conducted Harman’s single-factor test to assess common method variance. An exploratory factor analysis (principal component analysis) of all scale items revealed that the first unrotated component accounted for 12.78% of the total variance, indicating that common method bias was not a serious concern.

## 3 Results

### 3.1 Participants’ characteristics

A total of 620 operating room nurses enrolled in this study, 616 (99.4%) participants completed the electronic questionnaire, and 573 valid questionnaires were included for analysis, with a valid questionnaire response rate of 93.0%. The ages of the respondents ranged from 21 to 59 with a mean age of 34.87 ± 7.19 years, and most were female (83.9%). There were 442 (77.1%) operating room nurses who knew about the privacy regulations and 378 (66.0%) had attended privacy protection training. The complete demographic and professional characteristics of the participants are summarized in Table 1.

**Table 1:** Descriptive characteristics of the participating OR nurses (*N* = 573).

Variables	Category	Frequency (*n*)	Percentage (%)
Sex	Male	92	16.1
	Female	481	83.9
Ethnicity	Han	535	93.4
	Non-Han	38	6.6
Age	< 25 years	40	7.0
	26 ~ 30 years	142	24.8
	31 ~ 35 years	140	24.4
	≥36 years	251	43.8
Age (Mean ± SD)	34.87 ± 7.19		
Education level	≤ College	67	11.7
	Bachelor	479	83.6
	Master	27	4.7
Professional title	Registered nurse	260	45.4
	Senior nurse	251	43.8
	≥Co-chief nurse	62	10.8
Administrative position	General nurse	462	80.6
	Charge nurse	68	11.9
	Lead nurse	43	7.5
Duration of working	1 ~ 2 years	51	8.9
	3 ~ 5 years	82	14.3
	6 ~ 10 years	129	22.5
	≥11 years	311	54.3
Awareness of privacy regulations	No	131	22.9
	Yes	442	77.1
Participation in patient privacy protection training	No	195	34.0
	Yes	378	66.0

### 3.2 The scores and Pearson correlation analysis of PPS, MSQ-R-CV, and NASA-TLX

The mean scores for mental workload, moral sensitivity, and patient privacy protection were 45.450 ± 14.826, 45.775 ± 6.053, and 82.384 ± 5.169, respectively. Mental workload was negatively correlated with patient privacy protection (*r* = −0.655, *p* < 0.001) and moral sensitivity (*r* = −0.575, *p* < 0.001). Moral sensitivity was positively correlated with patient privacy protection (*r* = 0.506, *p* < 0.001). All shown in Table 2.

**Table 2:** Descriptive statistics and Pearson correlations for the main study variables (*N* = 573).

Variables	Mean	SD	1	2	3
1. Mental workload (NASA-TLX)	45.450	14.826	1		
2. Moral sensitivity (MSQ-R-CV)	45.775	6.053	−0.575^***^	1	
3. Patient privacy protection (PPS)	82.384	5.169	−0.655^***^	0.506^***^	1

NASA-TLX, National Aeronautics and Space Administration Task Load Index; MSQ-R-CV, Moral Sensitivity Questionnaire-Revised Chinese Version; PPS, Patient Privacy Scale.

***p < 0.001.

### 3.3 Mediating effect of moral sensitivity on the relationship between mental workload and patient privacy protection

**Figure 1:**
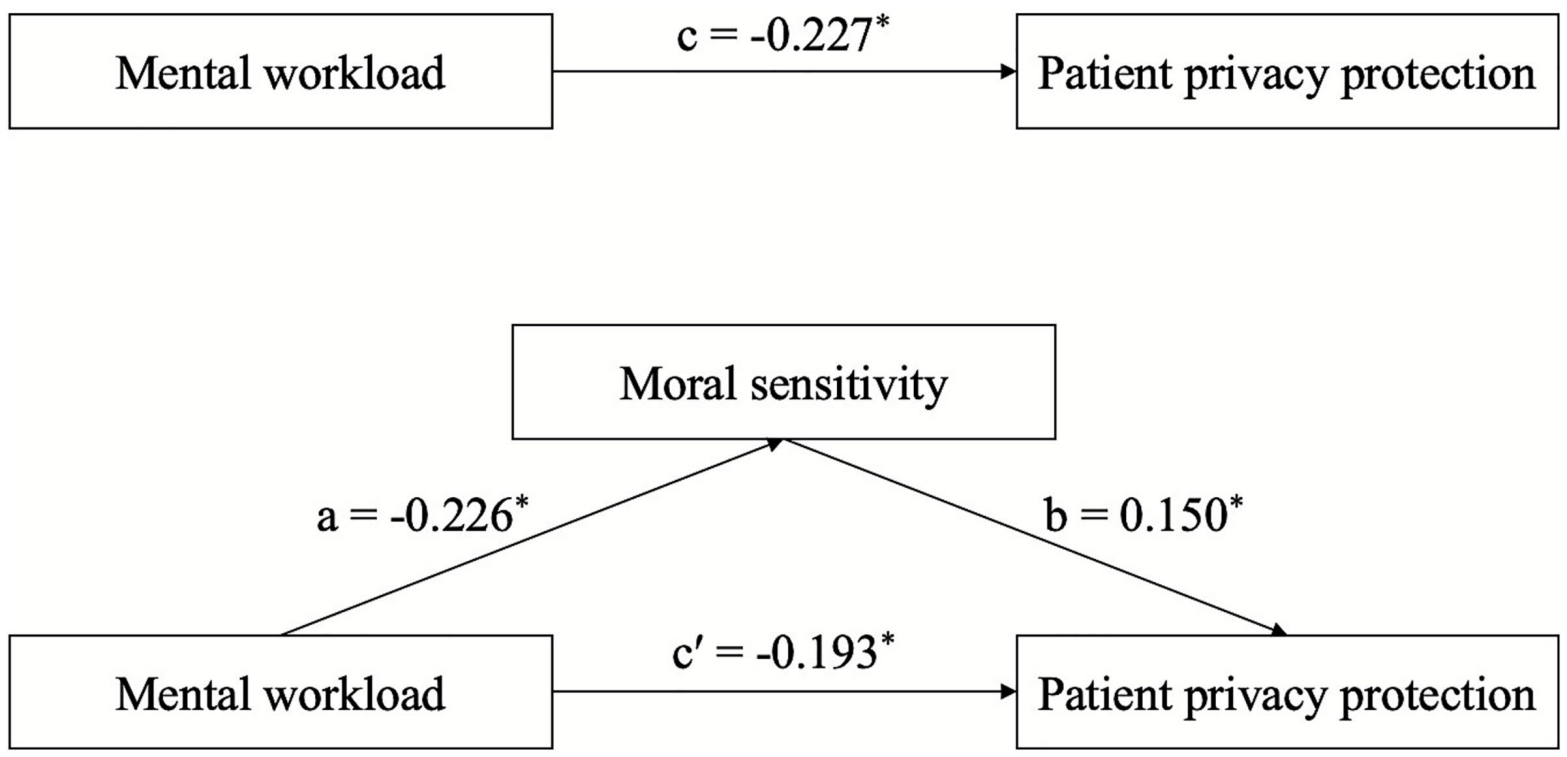
Diagram of the mediation effect model.

The results of the mediation analysis, outlining the specific paths, are presented in Figure 1 and Table 3. Path a represents the effect of mental workload (independent variable) on moral sensitivity (mediator). Path b represents the effect of moral sensitivity on patient privacy protection (dependent variable), controlling for mental workload. Path c represents the total effect of mental workload on patient privacy protection. Path c′ represents the direct effect of mental workload on patient privacy protection, after accounting for the indirect effect through moral sensitivity.

**Table 3:** Results of the bootstrap mediation effect test (*n* = 573).

Effect	Path	*β*	*SE*	LLCI	ULCI	Utility percentage
Total effect	NASA ⇒ PPS	−0.227	0.011	−0.249	−0.205	/
Direct effect	NASA ⇒ PPS	−0.193	0.013	−0.219	−0.167	85.02
Indirect effect	NASA ⇒ MSQ ⇒ PPS	−0.034	0.015	−0.067	−0.010	14.98

*β*, unstandardized coefficient; SE, standard error; LLCI refers to the lower limit of the 95% confidence interval for the estimated value, and ULCI refers to the upper limit of the 95% confidence interval for the estimated value.

After controlling for these potential confounders, the results showed that the total effect (path c) between mental workload and patient privacy protection was −0.227 (95% CI: −0.249 to −0.205), indicating that higher mental workload was associated with lower patient privacy protection. The coefficient for the relationship between mental workload and moral sensitivity (path a) was −0.226 (95% CI: −0.254 to −0.200), while the coefficient between moral sensitivity and patient privacy protection (path b) was 0.150 (95% CI: 0.086 to 0.213). The indirect effect (a * b) was −0.034 (95% CI: −0.067 to −0.010), which was statistically significant. The direct effect (path c′) between mental workload and patient privacy protection was −0.193 (95% CI: −0.219 to −0.167). The proportion of the indirect effect relative to the total effect, calculated as (a * b)/c = −0.034/−0.227 = 14.98%, indicating that moral sensitivity partially mediates the relationship between mental workload and patient privacy protection. The findings confirm the study’s primary hypothesis that moral sensitivity partially mediates the relationship between mental workload and patient privacy protection (see Figure 1 and Table 3 for further details).

## 4 Discussion

The findings of our study support the hypotheses that mental workload is negatively associated with patient privacy protection and moral sensitivity and that moral sensitivity serves as a mediator in the relationship between patient mental workload and patient privacy protection among operating room nurses in China.

The results showed that the scores of PPS among operating room nurses were in the upper-middle range when compared to the total scale score, suggesting that nurses demonstrate a commendable level of competence in protecting the privacy of surgical patients. 77.1% participants had the knowledge of privacy laws and regulations, and 66.0% respondents attended the training of patient privacy protection, which may contribute to improving the ability of safeguarding privacy for operating room nurses. Similarly, the mean score of mental workload in this study was in the moderate level, lower than those observed among nurses working in intensive care units (28), yet it still represented a significant concern as high mental workload has been consistently linked to adverse nurse outcomes in large-scale studies (29). As mental workload was influenced by individual-related factors, such as age and education level (30). Younger and less experienced nurses tend to be less familiar with nursing procedures and exhibit lower confidence in managing nursing events, which leads to a high level of mental workload (31). However, only 40 nurses who aged less than 25 and 67 nurses who held a college degree enrolled in our study, which may be the reason for the moderate level of mental workload. Furthermore, the MSQ-R-CV scores in this study population were slightly higher than those reported for the nurses in adult intensive care units by Pang et al. (32), yet lower than those observed in the study conducted by Guo et al. (33). Moral sensitivity is the foundation of moral activities. Thus, from the perspective of this study, it is essential to further enhance moral sensitivity and strengthen patient privacy protection practices among operating room nurses, while simultaneously implementing targeted strategies to reduce their mental workload.

Beyond these descriptive findings, the core relationships proposed in our theoretical model were strongly supported by the data. The results showed a positive correlation between patient privacy protection and moral sensitivity, aligning with findings from previous studies (3, 19). As the foundational element for moral behavior and moral capacity, and constituting a core component of ethical behavior, moral sensitivity played a crucial role in facilitating the recognition, development, and execution of moral behavior (34, 35). Sepehrirad et al. (3) found that moral sensitivity demonstrated a positive predictive relationship with the patient privacy protection, as evidenced by the fact that nurses with higher moral sensitivity place greater emphasis on protecting patients’ privacy rights. Furthermore, moral sensitivity has been demonstrated to be associated with job satisfaction and ethical decision-making among nurses, as well as the quality of nursing care for patients (7, 36, 37). Therefore, interventions can be implemented to enhance nurses’ moral sensitivity, which in turn may contribute to improved patient well-being.

The mediation effect model indicated that mental workload could directly influence patient privacy protection and could also indirectly impact patient privacy protection through moral sensitivity. Due to the intensive work-related stress and additional challenges, such as extended working hours, fast-paced environment, as well as personal management, operating room nurses suffered from excessive workload and even experienced occupational burnout (38). The substantial work pressure not only adversely affected their mental health, but also contributed to missed nursing care (15, 39). A study reported that physical workload was significantly associated with mental workload (13). As the physical workload of operating room nurses increases, their mental workload also rises, leading to an immediate emergence of nursing deficiencies. This may account for the direct association of mental workload on the patient privacy protection.

Mental workload was negatively associated with moral sensitivity in our study, which was different from the findings reported by Zahednezhad et al. (40) for nursing professionals working in critical care unit. This discrepancy may be attributable to key differences in the clinical contexts and sample characteristics. Firstly, the operating room environment is characterized by unique pressures, such as the management of complex, fast-paced surgical procedures and the responsibility for the vulnerable, anesthetized patient, which might intensify the depleting effect of high workload on cognitive resources available for ethical discernment. Secondly, cultural differences in work values and coping mechanisms between Chinese and the sample population of Zahednezhad et al.’s (40) study might influence how workload is perceived and its subsequent impact on moral sensitivity. Finally, differences in the measurement tools or the specific dimensions of workload captured could also contribute to the divergent findings. The mental workload was deemed as the most important factor in reducing moral sensitivity by nursing staff (41). Thus, higher mental workload among operating room nurses is associated with lower moral sensitivity. We recommend that nursing managers implement timely and targeted interventions to alleviate operating room nurses’ workload while simultaneously enhancing their moral sensitivity, thereby ensuring the safety of patient privacy. Furthermore, the results of this study indicate that moral sensitivity partially mediates the association between mental workload and patient privacy protection. These findings offer important implications for nursing management and clinical practice. This suggests that, in addition to moral sensitivity, other variables may serve as potential mediators in the relationship between mental workload and patient privacy protection. Consequently, further research is warranted to explore the underlying mechanisms through which mental workload influences the safeguarding of patient privacy.

Despite its contributions, this study must be interpreted in light of several limitations. A major strength of our study is that it is the first to investigate the relationship between mental workload, moral sensitivity, and patient privacy protection among operating room nurses. It is also the first to examine moral sensitivity as a mediating factor between mental workload and patient privacy protection. However, there are several limitations in our study. Firstly, the cross-sectional design precludes causal inference. The possibility of reverse causality (e.g., nurses who frequently encounter privacy dilemmas may experience higher mental workload) cannot be ruled out, and unmeasured confounders (e.g., team climate, hospital leadership style) might influence the observed relationships. Secondly, the collection of all primary data through self-reported questionnaires may introduce social desirability bias (e.g., overreporting privacy protection behaviors) and recall bias (e.g., inaccuracies in assessing workload). Future research could incorporate multi-source data, such as field research (42) or patient evaluations, to enhance objectivity. Finally, despite recruiting participants from four cities in China, the use of convenience sampling and the exclusive recruitment from tertiary hospitals may lead to selection bias. The findings primarily reflect the context of large urban teaching hospitals, which may differ from primary or rural healthcare settings in terms of workload and training resources, thus limiting the generalizability of the results to a broader population of healthcare institutions (43). Future research could expand the scope of the investigation to enhance the external validity of the findings.

## 5 Conclusion

This study reveals the relationship between mental workload and patient privacy protection among operating room nurses, and examines the mediating role of moral sensitivity in this association. These findings suggest that interventions aimed at addressing high mental workload may be associated with enhanced moral sensitivity and more frequent privacy protection behaviors among operating room nurses.

## Data Availability

The original contributions presented in the study are included in the article/supplementary material, further inquiries can be directed to the corresponding authors.
